# Risk Factors for Sporadic Domestically Acquired *Campylobacter* Infections in Norway 2010–2011: A National Prospective Case-Control Study

**DOI:** 10.1371/journal.pone.0139636

**Published:** 2015-10-02

**Authors:** Emily MacDonald, Richard White, Ricardo Mexia, Tone Bruun, Georg Kapperud, Heidi Lange, Karin Nygård, Line Vold

**Affiliations:** 1 Department of Infectious Disease Epidemiology, Norwegian Institute of Public Health, Oslo, Norway; 2 European Programme for Intervention Epidemiology Training (EPIET), European Centre for Disease Prevention and Control, Stockholm, Sweden; 3 Department of Health Statistics, Norwegian Institute of Public Health, Oslo, Norway; 4 Division of Infection Control, Norwegian Institute of Public Health, Oslo, Norway; USDA-ARS-ERRC, UNITED STATES

## Abstract

**Background:**

Campylobacteriosis is the most frequently reported food- and waterborne infection in Norway. We investigated the risk factors for sporadic *Campylobacter* infections in Norway in order to identify areas where control and prevention measures could be improved.

**Methods:**

A national prospective case-control study of factors associated with *Campylobacter* infection was conducted from July 2010 to September 2011. Cases were recruited from the Norwegian Surveillance System of Communicable Diseases (MSIS). Controls were randomly selected from the Norwegian Population Registry. Cases and controls were mailed a paper questionnaire with a prepaid return envelope. Univariable analyses using logistic regression were conducted for all exposures. A final parsimonious multivariable model was developed using regularized/penalized logistic regression, and adjusted odds ratios were calculated.

**Results:**

A total of 995 cases and 1501 controls were included in the study (response proportion 55% and 30%, respectively). Exposures that had significant increases in odds of *Campylobacter* infection in multivariable analysis were drinking water directly from river, stream, or lake (OR: 2.96), drinking purchased bottled water (OR: 1.78), eating chicken (1.69), eating meat that was undercooked (OR: 1.77), eating food made on a barbecue (OR: 1.55), living on a farm with livestock (OR: 1.74), having a dog in the household (OR: 1.39), and having household water supply serving fewer than 20 houses (OR: 1.92).

**Conclusions:**

Consumption of poultry and untreated water remain important sources of *Campylobacter* infection in Norway, despite ongoing control efforts. The results justify the need for strengthening education for consumers and food handlers about the risks of cross-contamination when preparing poultry and with consuming raw or undercooked chicken. The public should also be reminded to take precautions when drinking untreated water in nature and ensure continued vigilance in order to protect and maintain the quality of water from small-scale water supply systems.

## Introduction

**Fig 1:**
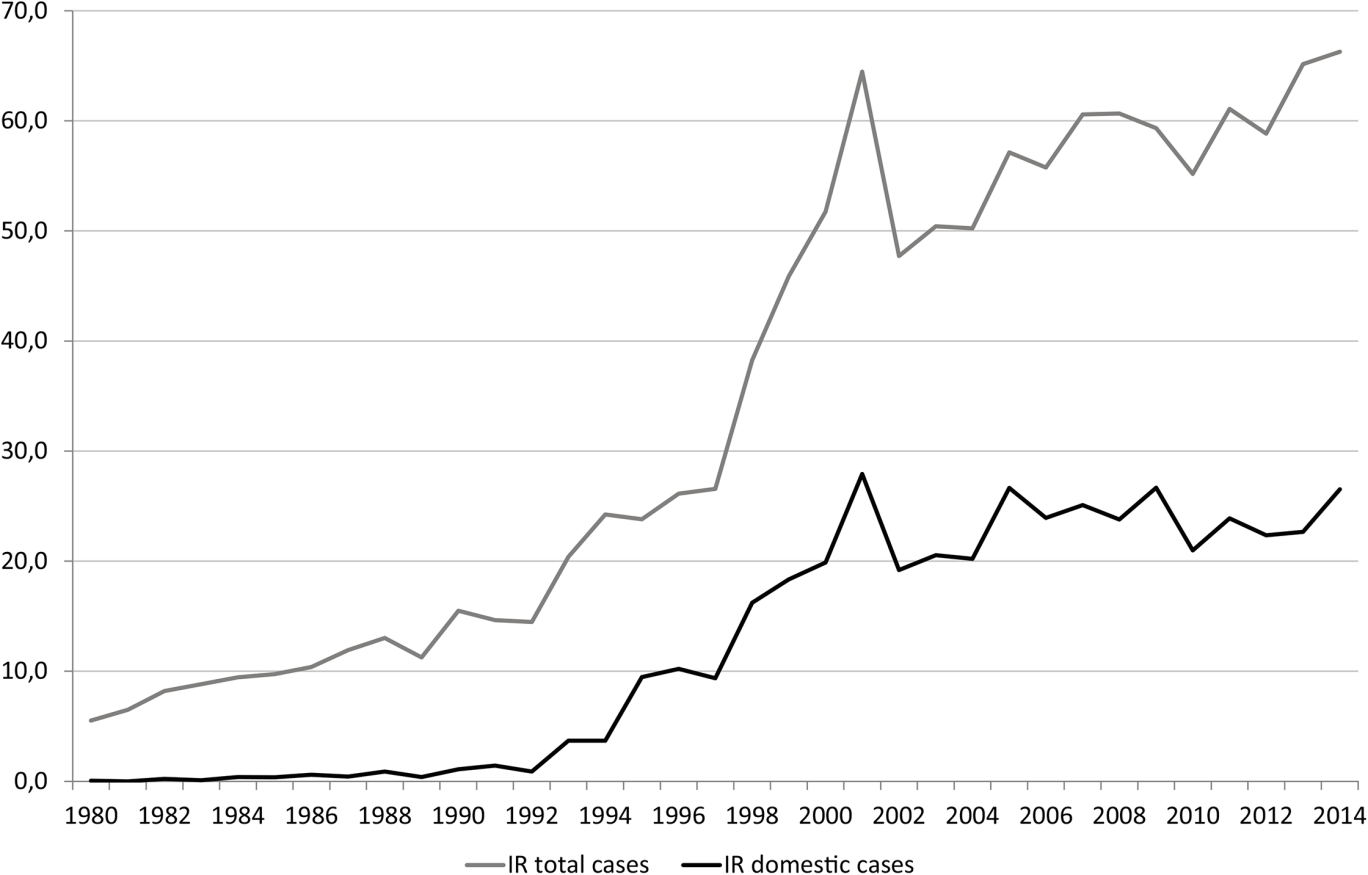
Annual Incidence Rates of *Campylobacter* Infections per 100 000 Population, 1980–2014 in Norway.

Campylobacteriosis is the most frequently reported food- and waterborne infection in Norway, as well as many other European countries [1]. Between 1990 and 2001, the annual notification rates of *Campylobacte*r infection in Norway via the Norwegian Surveillance System for Communicable Diseases (MSIS) increased substantially, peaking in 2001 at 65 cases per 100,000 population (Fig 1). Since 2001, the annual notification rates have continued to increase moderately, with between 2300 and 3000 cases reported annually, of which between 50 and 55% were associated with travel abroad. The notification rates for domestically-acquired *Campylobacter* have remained relatively stable, with an average incidence rate of 23.4 cases per 100,000 population reported between 2000 and 2013. Although symptoms of campylobacteriosis are generally limited to abdominal pain and diarrhea for several days, sequelae including Guillain-Barré syndrome (GBS), reactive arthritis, and irritable bowel syndrome (IBS) can also occur, causing considerable morbidity and economic impact [2]. Up to one-third of cases of GBS, which has a case-fatality rate between 3% and 10% in high-income countries, have been attributed to *Campylobacter* infection. More than 35% of patients with campylobacteriosis have reported IBS within 1–2 years after infection.

In Norway, outbreaks of campylobacteriosis have been associated with consumption of untreated or contaminated drinking water, unpasteurized milk, and lamb, as well as contact with farm animals and the butchering, preparation, and consumption of poultry [3]. Several large outbreaks have also occurred following bicycle races, linked to the participants’ exposure to mud [4]. However, most reported cases are sporadic and without an apparent source of infection [5]. In order to identify exposures associated with campylobacteriosis in Norway, three case-control studies have been previously conducted in different regions of the country. A case-control study conducted in three counties of Western Norway in 1999–2000 found that drinking untreated water, eating at barbecues, eating poultry bought raw, occupational exposure to animals, and eating undercooked pork were associated with *Campylobacter* infection, while eating mutton, eating fruit or berries, and recreational swimming were associated with reduced risk of illness [6]. A study from 1989–1990 in three counties of southeastern Norway found that consumption of sausages at a barbecue, daily contact with a dog, and eating poultry bought raw were associated with illness in multivariable analysis [7], while a study conducted in 1991–1994 in central Norway found that consumption of untreated drinking water and contact with dogs were associated with illness in multivariable analysis [8]. These results are supported by case-control studies conducted in a number of European countries, which have shown that sporadic *Campylobacter* infection is frequently associated with consumption of red meat and poultry, particularly when undercooked or barbecued, consumption of unpasteurized milk, contact with pets and farm animals, and eating at restaurants [9–17].

Since the previous Norwegian case-control studies, there have been several measures put in place to reduce the risk of acquiring *Campylobacter* infection from the water supply and from broiler products. In 2000, a government-initiated program for upgrading waterworks was introduced, resulting in a decrease in the number of waterworks and households who get their water from surface sources that are not disinfected [18]. In 2006–2007, the Norwegian Food Safety Authority conducted a nationwide audit of drinking water supply systems to assess water quality, quantity, and security throughout the country [19]. Over 350 Norwegian water supply systems (26%) for more than 20 households, supplying more than 2.8 million residents, were audited. Over 900 failures were identified, including lack of authorization (n = 64), missing hygienic barriers (n = 29), water not meeting current quality requirements for drinking water (n = 61), and water not disinfected after repairs to pipelines (n = 196). Since the audits, water supply owners have been required to make improvements where necessary and ensure the system’s authorization is up to date.

To reduce *Campylobacter* in broilers, several measures including sampling of flocks, advisory services for farms delivering positive flocks, and surveys at the retail level, were consolidated in an Action Plan in 2001 [20], which has been revised annually. Flocks are sampled prior to slaughter and chicken from flocks that are positive for *Campylobacter* are heat-treated or frozen following slaughter. In the first years after the implementation of the Action Plan, there was a considerable reduction in flocks positive for *Campylobacter* spp from 6.3% to 3.3% between 2002 and 2004 [21]. Since then, the percent of positive flocks has remained stable between 4% and 6% [22]. Simultaneously, the consumption of poultry has increased substantially in Norway, increasing from 4.6 kg per person per year in 1989 to 17.5 kg per person per year in 2011 [23]. Although the number of contaminated flocks at market has been almost halved, the number of chickens in each flock has nearly doubled. Nevertheless, due to measures implemented in the Action Plan, the number of broiler carcasses contaminated with *Campylobacter* at retail sale has not increased.

Despite these interventions, the incidence of *Campylobacter* infections in humans has not decreased correspondingly. There is therefore a need to identify the present risk factors to determine whether consumption of poultry products and water continue to be exposures associated with campylobacteriosis in Norway. The objective of this study was therefore to investigate risk factors for sporadic *Campylobacter* infections in Norway in order to identify areas where control and prevention measures could be improved. This case-control study was the first nationwide investigation of domestically acquired sporadic campylobacteriosis in Norway and the first study after the monitoring and control program for *Campylobacter* in broilers was implemented in broilers and nationwide audit of drinking water supply systems were implemented in 2001 and in 2006–2007, respectively.

## Methods

### Study design

A national prospective case-control study of factors associated with *Campylobacter* and *Salmonella* infections was conducted from July 2010 to September 2011. This period was selected to include a pilot period of three months (July 2010 to August 2010), followed by a full calendar year. This article presents the results of the *Campylobacter* study only. A case was defined as a resident of Norway with laboratory-confirmed campylobacteriosis caused by any species reported to MSIS from July 2010 to September 2011. *Campylobacter* infections are notifiable by both clinicians and laboratories in Norway. All cases reported to MSIS during the study period with postal address available were included in the study. Controls were randomly selected from the Norwegian Population Registry, a continuously updated registry of all residents of Norway. Four hundred unmatched controls were selected on a monthly basis. Participants were excluded if they reported history of international travel in the 14 days prior to onset of symptoms (for cases) or questionnaire completion (for controls). Controls were also excluded if they reported gastrointestinal illness (three or more loose stools within 24 hours or at least three of the following symptoms: vomiting, nausea, abdominal pain or fever) in the four weeks prior to questionnaire completion. A pilot study was conducted from July 2010 to September 2010. As few changes were made to the questionnaire following the pilot period, cases and controls interviewed during the pilot phase of the study were considered to be sufficiently similar to those from the designated study period to be included in the final study population.

### Questionnaires

Cases and controls were mailed a paper questionnaire with a prepaid return envelope. Participants were asked to return the questionnaire by mail, or answer the questions online using an electronic version of the questionnaire. Questionnaires were sent to cases on a weekly basis. For controls, the monthly number of controls (n = 400) was divided by number of working days so that questionnaires were sent out on continual basis (approximately 20 questionnaires per working day). The questionnaire was accompanied by a cover letter which explained that participation was voluntary, that the data collected through the study would be treated as confidential and that participants could withdraw from the study at any stage. If a response had not been received after two weeks, up to three attempts to contact cases and controls were made by telephone to encourage participation. Potential respondents were offered the option of responding to the questionnaire by telephone. If a case or control refused to participate, no further calls were made. If a completed questionnaire was not returned and attempts to contact the case or control were unsuccessful, the case or control was considered a non-respondent. If a case or control was under the age of 16, the questionnaire was sent to a parent of the child who was asked to help the child complete the questions. Submission of the completed questionnaire was considered consent for participation. This study received ethical approval from the Regional Ethical Committee for South East Norway.

### Exposures

Fifty-eight broad yes/no questions concerning risk factors were asked in the questionnaire, mostly concerning activity of the week before (e.g. “During the last week, did you eat chicken?”). If a respondent answered affirmatively to a question, they were directed to further derivative questions concerning frequency of the activity and yes/no circumstance-specific concerns (e.g. “Was the purchased chicken raw and frozen?” and “Was the chicken purchased ready-made?”). For data cleaning purposes, “uncertain” responses were coded as missing.

### Confounders

We considered sex (male/female), age (continuous years), number of people in house (continuous), county (18 dummy variables representing 19 counties), and education (4 dummy variables representing 5 levels: not completed primary school, primary to middle school, high school, university, and other) as potential confounders. Age and number of people in house were specified as continuous variables due to a desire for a parsimonious model, as manipulation of continuous variables (e.g. changing cutoffs into categories, or position of spline knots) can unconsciously lead to a biased model. After model fitting, residuals were assessed to ensure linearity assumptions were not violated.

### Statistical analyses

Adjusting for the above listed potential confounders, we ran 58 separate logistic regression analyses on the broad risk factors, accounting for multiple testing by using the Bonferroni correction. As supplemental information, these analyses were rerun in two strata: restricting to summer (June, July, August) and not-summer. We then ran supplemental logistic regression analyses in the derivative risk factor questions. When the questions were about frequency of risk-factor occurrence, the entire cohort was analysed. When the questions were about more specific aspects of the original broad risk factor, the analysis cohort was restricted to only those who answered yes to the original broad risk factor. Controls were used as the comparison group for all analyses.

We then constructed a multivariable model to investigate the associations between broad risk factors and risk of *Campylobacter* infection, when accounting for the relationships between the broad risk factors. We included all 58 broad risk factors (plus potential confounders) as explanatory variables in our multivariable model. Due to the large number of explanatory variables we used BOLASSO to construct a parsimonious model [24]. Briefly, missing data was accounted for by using multiple imputations using regularized regression (LASSO). For each of the ten imputed datasets, 200 bootstrapped datasets were constructed and LASSO penalization using 20-folds cross-validation selected a parsimonious model. Variables selected in all 2000 LASSO regressions were then included in the final model–an ordinary logistic regression using the imputed datasets. The population attributable fraction (PAF) was calculated for the multivariable model, approximating the risk ratio with the odds ratio. Post hoc-analysis for interaction between age and type of animal contact was performed, as well as post hoc complete-case sensitivity analyses, in which variables relating to chronic illness (including gastrointestinal diseases, food allergies/intolerances and immunocompromising diseases) and use of medications (such as antacids, diabetes medication, cortisone/steroids and vitamins/supplements) were included.

## Results

During the 15-month study, 4379 cases of campylobacteriosis were notified via MSIS. Of these, 3397 cases were not reported as infected abroad, had valid Norwegian postal addresses, and were sent the questionnaire. Responses to the questionnaire were received from 1865 cases, corresponding to a response proportion of 55%. Questionnaires were sent to 5808 controls, of which 1738 responded, corresponding to a response proportion of 30%. We subsequently excluded any respondent who had been outside of Norway in the previous two weeks, corresponding to 870 cases and 110 controls, leaving 995 cases and 1628 controls. We then excluded 127 controls with (unspecified) gastrointestinal illnesses, leaving 995 cases and 1501 controls, for a total of 2496 subjects. Of the 995 cases included in the study, 73.0% were infected with *C*. *jejuni* (n = 726), 0.6% were *C*. *coli* (n = 6), 1.4% were other species of pathogenic *Campylobacter* (n = 14) and 25.0% (n = 249) did not have the species indicated. When the participating controls are compared to the Norwegian population, males are underrepresented (44.5% of controls compared to 50.3% of the population, p = 0.002) and the study participants are older than the general population (p<0.001). The participant and non-participant cases did not differ significantly in terms of sex and age group.

The mean age of cases and controls was 40.9 (95% CI: 39.6–42.1) and 42.7 (95% CI: 21.6–43.8), respectively. Male subjects comprised 49.4% of cases and 44.5% of controls. Cases belonged to larger households and reported higher education levels than controls (Table 1). Cases reported location of residence in Western Norway, and in the counties of Agder, Rogaland, Hedmark, Oppland, and Trøndelag more frequently than controls.

**Table 1:** Demographic characteristics of cases (n = 995) and controls (n = 1501).

Variable	Cases, n (%)	Controls, n (%)	P value^#^
*Sex*			
Male	500 (50.6)	824 (55.5)	
Female	488 (49.4)	662 (44.5)	0.018
*Age group*			
0 to 9	99 (10.1)	147 (10.0)	
10 to 19	59 (6.0)	138 (9.4)	
20 to 39	289 (29.6)	316 (21.6)	
40 to 59	333 (34.1)	493 (33.7)	
60 to 94	197 (20.2)	369 (25.2)	<0.001
*People in household*			
Live alone	97 (9.8)	195 (13.1)	
Two people	355 (35.9)	531 (35.7)	
3 to 5 people	508 (51.4)	704 (47.4)	
6 or more people	29 (2.9)	56 (3.8)	0.034
*Education level completed*			
Primary school not completed	110 (11.4)	233 (15.8)	
Primary school or middle school	142 (14.7)	222 (15.1)	
High school	342 (35.3)	465 (31.5)	
University	341 (35.2)	499 (33.9)	
Other	34 (3.5)	55 (3.7)	0.024
*Geographical region*			
Oslo and Akershus	189 (19.0)	381 (25.4)	
Hedmark and Oppland	99 (9.9)	112 (7.5)	
Southeast Norway	162 (16.3)	303 (20.2)	
Agder and Rogaland	183 (18.4)	194 (12.9)	
Western Norway	200 (20.1)	240 (16.0)	
Trøndelag	99 (9.9)	126 (8.4)	
Northern Norway	63 (6.3)	144 (9.6)	<0.001

^**#**^ P values shown are for Pearson's Chi-Squared test, testing percentages ignoring missing.

### Univariable analysis

#### Food exposures

After correction for multiple testing, we found several meat-related exposures that significantly increased the odds of *Campylobacter* infection in univariable analysis (Table 2): eating food made on a barbecue (OR 1.93; 95% CI: 1.59–2.34), eating undercooked meat (OR 1.71; 95% CI: 1.28–2.27), and eating chicken (OR 1.67; 95% CI: 1.37–2.04). Cases were not more likely than controls to report consumption of beef, ground meat, cold cuts or cured meats, while consumption of pork, turkey, and lamb/mutton were associated with a reduced risk of illness.

**Table 2:** Univariable logistic regression of food and water risk factors for campylobacteriosis.

Exposure		Cases exposed N (%)	Controls exposed N (%)	OR (95% CI) Crude model	OR (95% CI) Final model^#^
**Water**					
	Drinking water directly from river, stream, or lake	106 (11)	60 (4)	2.84 (2.04, 3.94)***	2.78 (1.97, 3.93)***
	Water supply for own house vs baseline of 20+ houses	135 (17)	102 (9)	2.17 (1.64, 2.85)***	2.13 (1.58, 2.88)***
	Drinking purchased bottled water	349 (43)	418 (30)	1.74 (1.45, 2.09)***	1.78 (1.47, 2.16)***
	Noticed something strange with home water	33 (4)	33 (2)	1.51 (0.92, 2.46)	1.64 (0.98, 2.74)
	Water supply for 1–19 houses vs baseline of 20+ houses	36 (5)	38 (3)	1.55 (0.97, 2.47)	1.58 (0.96, 2.61)
	Swam in the sea, freshwater, outdoor hot tub, or pool	131 (14)	194 (14)	0.99 (0.78, 1.26)	1.06 (0.82, 1.37)
	Drinking tap water not at home	594 (69)	916 (67)	1.12 (0.93, 1.34)	1.02 (0.83, 1.25)
	Drinking water from a dispenser	144 (16)	232 (17)	0.92 (0.73, 1.16)	0.78 (0.61, 0.99)
	Treated drinking water at home (chlorine or UV filter)	415 (73)	704 (80)	0.66 (0.52, 0.85)+	0.65 (0.49, 0.85)+
	Drinking tap water at home	910 (94)	1362 (97)	0.46 (0.31, 0.68)**	0.43 (0.28, 0.66)**
**Meat**					
	Eating food made on a grill	348 (39)	361 (26)	1.87 (1.56, 2.24)***	1.93 (1.59, 2.34)***
	Eating undercooked meat	110 (14)	119 (9)	1.74 (1.32, 2.30)**	1.71 (1.28, 2.27)*
	Eating chicken	547 (69)	791 (59)	1.57 (1.31, 1.89)***	1.67 (1.37, 2.04)***
	Eating cold cuts	604 (69)	946 (69)	1.03 (0.86, 1.24)	1.04 (0.86, 1.26)
	Eating ground meat	743 (88)	1206 (87)	1.06 (0.82, 1.37)	0.95 (0.72, 1.25)
	Eating cured meats	537 (62)	879 (64)	0.92 (0.77, 1.10)	0.93 (0.77, 1.12)
	Eating beef	419 (50)	714 (52)	0.92 (0.78, 1.09)	0.88 (0.73, 1.05)
	Eating pork	435 (54)	863 (63)	0.68 (0.57, 0.82)**	0.70 (0.58, 0.85)*
	Eating turkey	39 (4)	92 (7)	0.62 (0.42, 0.91)	0.64 (0.43, 0.96)
	Eating foreign bought meat	132 (15)	324 (24)	0.56 (0.45, 0.70)***	0.64 (0.51, 0.82)*
	Eating mutton/lamb	101 (11)	278 (20)	0.51 (0.40, 0.65)***	0.51 (0.39, 0.66)***
**Other foods**					
	Drinking unpasturised milk	31 (4)	30 (2)	1.58 (0.95, 2.63)	1.35 (0.79, 2.32)
	Eating raw berries	418 (47)	601 (44)	1.14 (0.97, 1.35)	1.25 (1.05, 1.50)
	Eating salad	693 (78)	1053 (76)	1.13 (0.92, 1.38)	1.03 (0.82, 1.28)
	Eating fresh herbs	207 (24)	409 (30)	0.72 (0.59, 0.88)+	0.76 (0.62, 0.94)
	Eating soft cheese	230 (26)	464 (34)	0.69 (0.57, 0.83)**	0.72 (0.59, 0.88)+
	Eating raw vegetables	680 (76)	1159 (83)	0.65 (0.53, 0.80)**	0.67 (0.54, 0.84)*
	Eating eggs	715 (82)	1206 (86)	0.73 (0.58, 0.92)	0.67 (0.52, 0.86)
	Eating asparagus	25 (3)	61 (4)	0.59 (0.37, 0.94)	0.54 (0.33, 0.89)
	Eating dried herbs	585 (71)	1113 (81)	0.58 (0.47, 0.71)***	0.54 (0.44, 0.67)***
	Eating raw fruit	822 (89)	1332 (95)	0.42 (0.30, 0.57) ***	0.45 (0.33, 0.63)***

^**#**^Adjusted for: Is male, Age, Number of people in house, County (dummy variables), Education (categorical). Answers for all variables were not available for all participants. Denominators in percentages vary.

Significance indicators: 0

*** 0.001

** 0.01

* 0.05

+ 0.1.

Indicators based on P values adjusted for multiple testing using Bonferroni correction.

In univariable derivative analysis (S1 Table), we found that within chicken eaters, buying frozen raw chicken was associated with lower odds of *Campylobacter* infection (OR 0.56; 95% CI: 0.44–0.73), while eating ready-made chicken from restaurant or takeaway was associated with higher odds of *Campylobacter* infection (OR 1.45; 95% CI: 1.12–1.88). A secondary analysis found that compared to not consuming *any* chicken, consumption of chicken purchased raw and unfrozen, and consumption of ready-made chicken, were associated with infection (OR 1.79; 95% CI: 1.42–2.26, and OR 2.22; 95% CI: 1.68–2.93, respectively) while consumption of previously frozen chicken was not associated with infection (OR 1.21; 95% CI: 0.93–1.58). A protective dose response was observed with frequency of purchase of frozen raw chicken (OR 0.76; 95% CI: 0.67–0.88).Within people who ate undercooked meat, eating poultry was associated with higher odds of *Campylobacter* infection (OR 8.01; 95% CI: 3.62–17.72). Within people who ate food prepared on a barbecue, eating poultry was associated with higher odds of *Campylobacter* infection (OR 1.95; 95% CI: 1.34 to 2.84). Several food exposures were found to be associated with reduced risk of infection in univariable analysis, including consumption of raw vegetables (OR 0.67 95%; CI: 0.54 to 0.84), dried herbs (OR 0.54; 95% CI: 0.44 to 0.67) and raw fruits (OR 0.45; 95% CI: 0.33 to 0.63). No significant differences in consumption of unpasteurized milk and salad were observed between cases and controls.

#### Water exposures

Drinking water directly from river, stream, or lake (OR 2.78; 95% CI: 1.97 to 3.93), having single-household water supply compared to a baseline of more than 20 houses (OR 2.13; 95% CI: 1.58 to 2.88), and drinking purchased bottled water (OR 1.78; 95% CI: 1.47 to 2.16) were significantly associated with *Campylobacter* infection in univariable analysis. We observed a dose-response association with glasses of purchased bottled water consumed per day (OR: 1.16; 95% CI: 1.09 to 1.22). Drinking tap water at home was found to significantly decrease the odds of *Campylobacter* infection (OR 0.43; 95% CI: 0.28 to 0.66). We did not observe a significant dose-response association with number of glasses of tap water drunk at home each day (OR 0.97; 95% CI: 0.93 to 1.01). Although swimming in general was not associated with illness, swimming in outdoor freshwater (OR 2.34; 95% CI:1.25 to 4.36) and swimming in the sea (OR 2.42; 95% CI 1.38–4.26) were associated with illness while swimming in indoor pools decreased the odds of illness (OR 0.23; CI: 0.13–0.42) in derivative univariable analysis. We did not find a significant interaction term between these water variables and children less than or equal to five years old.

#### Animal, environmental and occupational exposures

Two animal exposures were found to be associated with illness (Table 3): living on a farm with livestock (OR 2.04; 95% CI: 1.43–2.91) and having a dog in the household (OR 1.56; 95% CI: 1.27–1.93). No significant associations were observed related to other animals or animal feces, including reptiles, hedgehogs, cats or wild birds. A multiplicative interaction term was introduced between children (under or equal to 5 years) and contact with animals or animal feces, showing that risk of *Campylobacter* associated with contact with dogs or dog feces was significantly increased in children (interaction OR 2.4; 95% CI 1.09–5.26), and borderline significant with respect to contact with cats or cat feces (interaction OR 2.4; 95% CI 0.99–5.85) or visiting a farm with livestock (interaction OR 2.21; 95% CI 0.92–5.35).

**Table 3:** Univariable logistic regression of animal, environmental and occupational risk factors for campylobacteriosis.

	Exposure	Cases exposed N (%)	Controls exposed N (%)	OR (95% CI) Crude model	OR (95% CI) Final model^#^
**Animal contact**					
	Live on a farm with livestock	96 (10)	62 (4)	2.43 (1.74, 3.38)***	2.04 (1.43, 2.91)**
	Dog in household	259 (27)	253 (19)	1.66 (1.36, 2.03)***	1.56 (1.27, 1.93)**
	Hedgehog in garden or neighbourhood	4 (0)	3 (0)	1.88 (0.42, 8.40)	1.55 (0.34, 7.03)
	Turtle, snakes, or other reptiles in household	4 (0)	3 (0)	1.88 (0.42, 8.40)	1.55 (0.34, 7.03)
	In contact with other animals or animal excrement	199 (21)	204 (15)	1.57 (1.27, 1.95)**	1.47 (1.16, 1.85)+
	Contact with turtle, snakes, or other reptiles	7 (1)	7 (1)	1.53 (0.53, 4.38)	1.46 (0.50, 4.29)
	Fed or had contact with hedgehog	7 (1)	7 (1)	1.53 (0.53, 4.38)	1.46 (0.50, 4.29)
	Visited a farm with livestock	133 (14)	135 (10)	1.54 (1.20, 1.99)*	1.38 (1.06, 1.81)
	Cat in household	244 (25)	268 (19)	1.40 (1.15, 1.70)*	1.32 (1.07, 1.62)
	Contact with dog or dog feces	313 (39)	442 (35)	1.21 (1.01, 1.45)	1.20 (0.99, 1.45)
	Contact with cat or cat feces	207 (27)	298 (24)	1.20 (0.97, 1.47)	1.10 (0.89, 1.37)
	Contact with wild birds or bird feces	72 (8)	115 (8)	0.96 (0.70, 1.30)	0.96 (0.69, 1.33)
**Hygiene**					
	Observed flies on food	34 (4)	41 (3)	1.36 (0.85, 2.16)	1.46 (0.90, 2.37)
	Frequently washes hands before eating food	358 (46)	483 (45)	1.08 (0.89, 1.30)	1.12 (0.92, 1.36)
	Frequently washes hands after bathroom	811 (86)	1158 (85)	1.12 (0.88, 1.41)	1.11 (0.87, 1.43)
	Children only: eaten snow, icicle, sand, dirt or played in sandbox	62 (6)	87 (6)	0.75 (0.54, 1.03)	1.02 (0.66, 1.59)
	Frequently washes hands after contact with animals or birds	331 (48)	501 (53)	0.82 (0.67, 1.00)	0.87 (0.70, 1.07)
	Frequently washes hands before making food	640 (74)	936 (75)	0.96 (0.79, 1.17)	0.97 (0.79, 1.19)
	Frequently washes kitchen tools between use on raw meat	680 (81)	1031 (85)	0.71 (0.56, 0.89)	0.71 (0.56, 0.91)
	Frequently washes hands after contact with raw meat	600 (74)	927 (80)	0.69 (0.55, 0.85)*	0.73 (0.58, 0.91)
**Occupational/ Location**					
	Attend or work in a kindergarten, park, or nursery	114 (12)	135 (9)	1.32 (1.01, 1.71)	1.57 (1.14, 2.16)
	Eat food made in a kindergarten, park, or nursery	98 (35)	118 (33)	1.11 (0.79, 1.54)	1.41 (0.90, 2.21)
	Live or work in retirement home, hospital, or other institution	110 (11)	158 (11)	1.07 (0.83, 1.38)	1.03 (0.78, 1.36)
	Eating food made at a restaurant	469 (51)	711 (51)	1.03 (0.88, 1.22)	0.94 (0.78, 1.12)
	Other person in household abroad in the last four weeks	111 (12)	215 (16)	0.70 (0.54, 0.89)	0.72 (0.56, 0.94)
	Work at a cafe, restaurant, or other serving place	22 (2)	41 (3)	0.81 (0.48, 1.37)	0.61 (0.35, 1.06)

^**#**^Adjusted for: Is male, Age, Number of people in house, County (dummy variables), Education (categorical). Answers for all variables were not available for all participants. Denominators in percentages vary.

Significance indicators: 0

*** 0.001

** 0.01

* 0.05

+ 0.1.

Indicators based on P values adjusted for multiple testing using Bonferroni correction.

#### Occupational, hygiene and health exposures

Potential exposures through contact with healthcare or food services were not significantly associated with infection. Working in a childcare facility was associated with illness (OR 1.57, 95% CI: 1.14–2.16). Behavioral practices, such as washing hands after having animal contact, before food preparation and after using the toilet, were not significantly associated with decreased risk of illness. Frequently washing kitchen utensils between use on raw meat and other food items (OR 0.71, 95% CI: 0.56–0.91) and washing hands after having contact with raw meat (OR 0.73, 95% CI: 0.58–0.91) were associated with reduced risk of infection. Having a family member return from a trip abroad in the preceding four weeks was associated with a reduced risk of illness (OR 0.72, 95% CI: 0.56–0.94). The point estimates of risk factors did not change substantially after including variables relating to chronic illness in post-hoc complete-case sensitivity analyses.

### Multivariable analysis

When placed in the same model, eight exposures had significant increases in odds of *Campylobacter* infection (Table 4): drinking water directly from river, stream, or lake (OR 2.96), having a water supply for less than 20 houses versus a baseline of 20 or more houses (OR 1.92), drinking purchased bottled water (OR 1.78), eating undercooked meat (OR 1.77), living on a farm with livestock (OR 1.74), eating chicken (OR 1.69), eating food made on a grill (OR 1.55), and having a dog in the household (OR 1.39). Frequently washing hands after having contact with raw meat was associated with a decreased risk of *Campylobacter* infection (OR 0.53) Based on the PAF, the most important factors associated with campylobacteriosis were eating chicken (30.3%), drinking bottled water (21.3%) and eating food made on a barbecue (14.6%).

**Table 4:** Multivariable BOLASSO regression of risk factors for campylobacteriosis.

Exposure	aOR	P-value	PAF (%)
Drinking water directly from river, stream, or lake	2.96 (2.09, 4.20)***	0.000	12.3
Water supply for 1–19 houses vs baseline of 20+ houses	1.92 (1.46, 2.53)***	0.000	12.4
Drinking purchased bottled water	1.78 (1.47, 2.15)***	0.000	21.3
Eating undercooked meat	1.77 (1.32, 2.36)***	0.000	7.5
Live on a farm with livestock	1.74 (1.19, 2.53)**	0.004	4.8
Eating chicken	1.69 (1.39, 2.06)***	0.000	30.3
Eating food made on a grill	1.55 (1.28, 1.88)***	0.000	14.6
Dog in household	1.39 (1.12, 1.72)**	0.003	7.9
Cat in household	1.21 (0.97, 1.49)+	0.087	4.3
Frequently washes hands after contact with raw meat	0.66 (0.53, 0.82)***	0.000	-

1. Multivariable BOLASSO logistic regression (odds ratio reported). 20-fold cross validation used for model selection.

2. Missing data was accounted for by multiple imputations using regularized regression (LASSO). 10 datasets were imputed and 100 bootstraps were then generated from each imputed dataset.3. PAF (Population attributable fraction) was calculated using odds ratios as approximations for risk ratios.

3. Significance indicators: 0

*** 0.001

** 0.01

+ 0.1.

## Discussion

In this first nationwide case-control study of risk factors for campylobacteriosis, we found that exposures in the following three broad categories were associated with illness: consumption of water (drinking untreated water, drinking bottled water and small scale household water supply), consumption of chicken and undercooked meat (eating chicken, eating undercooked meat, and eating food cooked on a grill) and contact with animals (living on a farm with livestock and having a dog in the household). Consumption of untreated water, poultry and undercooked meat, as well as contact with animals are all exposures commonly reported as associated with campylobacteriosis [25]. In addition, many of these findings are consistent with the results of the previous Norwegian case-control studies conducted between 1989 and 2000, despite using different models and study designs in different parts of the country (Table 5). This suggests that the most common exposures to *Campylobacter* in Norway remain present, despite control efforts in several areas.

**Table 5:** Summary of results from case-control studies on risk factors for campylobacteriosis in Norway.

Dates of study	Location of study	Description of study	Exposures associated with campylobacteriosis in multivariable analysis	Exposures associated with reduced risk of campylobacteriosis in multivariable analysis
1989–1990 [7]	Southeastern Norway (Oslo, Akershus and Buskerud)	Inclusion of 52 cases and 103 controls matched by age, sex and geographical location; Analysis with conditional logistic regression	Consumption of sausages at a barbecue (OR = 7.64; CI 1.83–31.89), daily contact with a dog (OR = 4.26; CI 1.21–15.01), eating poultry brought into the house raw (OR = 3.20; CI 1.17–8.76)	None.
1991–1994 [8]	Central Norway (Trøndelag County)	Inclusion of 56 cases and 117 controls matched by sex, age and geographical location; Analysis with conditional logistic regression	Consumption of untreated water (OR = 12.3; CI 3.94–38.1), proximity to a dog (OR 3.71; CI 1.51–9.10)	None.
1999–2000 [6]	Western Norway (Rogaland, Hordaland and Sør-Trøndelag)	Inclusion of 212 cases and 422 controls matched by sex, age and geographical location; Analysis with conditional logistic regression	Drinking untreated water (OR = 2.5; CI 1.2–5.4), eating at barbecues (OR = 4.1; CI 1.7–9.9), eating poultry bought raw (OR = 1.4; CI 1.0–2.0), having occupational exposure to animals (OR = 19.3; CI 1.7–222.3), eating undercooked pork (OR = 37.0; CI 1.7–830.9)	Eating mutton (OR = 0.4; CI 0.2–0.8), Eating raw fruits or berries (OR = 0.9; CI 0.9–1.0), swimming in a lake/sea/pool (OR = 0.7; CI 0.5–1.0)
2010–2011	Nationwide survey	Inclusion of 995 cases and 1501 randomly selected controls; Analysis with regularized/penalised logistic regression	Drinking water directly from river, stream, or lake (OR 2.96), having a water supply for less than 20 houses vs baseline of 20+ houses (OR 1.92), drinking purchased bottled water (OR 1.78), eating undercooked meat (OR 1.77), living on a farm with livestock (OR 1.74), eating chicken (OR 1.69), eating food made on a grill (OR 1.55), and having a dog in the household (OR 1.39)	Frequently washing hands after touching raw meat (OR = 0.66; CI 0.54–0.80)

Following the implementation of the *Campylobacter* Action Plan, the notification rate for campylobacteriosis remains unchanged and consumption of chicken is still an important source of infection. However, although the consumption of chicken among the Norwegian population increased substantially in the last twenty years, the number of *Campylobacter* contaminated chicken carcasses at retail sale has remained fairly stable. Although efforts to reduce *Campylobacter* contamination in broilers do not appear to have led to a significant reduction in illness among humans, it is possible that the number of *Campylobacter* contaminated chicken carcasses on the market would have been even higher in absence of the Action Plan. Enhanced efforts on encouraging practices over which consumers have control, including washing hands frequently after handling raw meat, avoiding cross-contamination when grilling and ensuring all meat is cooked thoroughly, may therefore play a larger role in reducing human illness.

Although consumption of chicken remains an important exposure for campylobacteriosis, which is a finding consistent with many other case-control studies, how chicken is stored and prepared leads to important differences in the risk of illness. Buying raw, frozen chicken was not associated with *Campylobacter* infection when compared to people who do not consume chicken. It is biologically plausible that reduced risk of infection can be associated with frozen chicken, as freezing substantially reduces the number of viable *Campylobacter* [26]. However, the thawing process must be properly controlled. Consuming chicken that is not fully cooked and chicken that was cooked on a barbecue were also associated with illness among people who ate chicken. The findings of the study suggest that guidance for the safe preparation of poultry at the household level should also be reinforced. The fact that illness among chicken consumers was more frequently associated with preparing food on barbecues and among people who eat undercooked meat reinforces that ensuring proper handling and heat treatment of raw chicken continues to present a challenge. This may be not only from exposure through consumption of undercooked meat, but more importantly through cross-contamination of other food items and utensils during preparation. Frequent washing of hands after having contact with raw meat was associated with a reduced risk of infection, and this is a hygiene practice that should be repeatedly promoted by food safety and public health authorities.

Several factors were associated with reduced risk of infection in univariable analysis, including consumption of raw fruits and vegetables, as well as pork and lamb/mutton. However, unlike handwashing, which is likely to directly reduce transmission of the infection, consumption of these food items is more likely to reflect dietary choices among controls. People that choose not to consume chicken may be more likely to eat other meats as an alternative. Additionally, people that have healthier lifestyles may have been more likely to participate in the study, leading to an overrepresentation of controls that choose diets that include more fruits and vegetables, and less red meat, than cases.

Buying ready-to-eat chicken was associated with higher odds of *Campylobacter* infection in the derivative analysis. A meta-analysis of the relative importance of risk factors for sporadic campylobacteriosis found that consumption of chicken in restaurants was an important source of infection [25]. Restaurants may present an increased risk due to differences in hygiene practices and preparation of food products compared to the home environment, including the potential for lack of care when preparing meats, inconsistency in temperatures for cooking and storage of meat products, and the possibility of cross-contamination between raw food items and ready-to-eat food items. These results suggest that there is a continued need to reinforce that food handlers ensure a high level of hygiene is maintained in restaurants and takeaways, particularly when handling or preparing poultry.

Drinking untreated water remains an important source of *Campylobacter* infection in Norway. This includes consuming water directly from untreated sources, such as rivers, streams or lakes, and consuming water from single household water sources. Untreated drinking water has been implicated in several *Campylobacter* outbreaks [27–30]and has been found as a risk factor in several case-control studies from other countries [13, 25, 31]. In addition, a recent study conducted on waterborne outbreaks in the Nordic countries found that the occurrence of heavy precipitation events increases the risk of occurrence of waterborne outbreaks in households with single-household water supplies (data in publication). Data indicates a significant reduction in the number of people in households serviced by surface water that is not disinfected from over 80 000 in 2001 to under 10 000 in 2011 [18]. However, this reduction applies to waterworks that supply more than 50 people.

In the present study, respondents were not asked to provide further information on where they had consumed untreated water, but it is likely that cabins, cottages or summer homes with wells or other single-unit water supply systems pose a particular risk as the water can be easily contaminated by animals and birds that have access to unprotected water sources. In the last case-control study from Norway [6], exposure to water from unprotected sources like cabins was reported by more than 50 percent of the cases who had consumed untreated water, thus contributing substantially to the PAF attributed to that exposure. It is therefore likely that the importance of untreated has been underestimated in the present study. Nevertheless, the results of this study reinforces that households with small-scale water supplies must be vigilant to avoid contamination and to consider treatment options, and that consumption of untreated water should be avoided. Appropriate water collection and storage options at cabins and summer homes should also be reiterated. In addition, people drinking water directly from lakes or rivers while engaging in outdoor activities should be aware of the risk of contamination and waterborne campylobacteriosis should be considered in patients presenting with gastroenteritis and a history of participation in recreation activities that involve contact with untreated water.

Consumption of bottled water was found to be associated with *Campylobacter* infection in multivariable analysis. This finding, which was supported by a dose-response relationship, was not previously identified as a risk factor in Norway and is not commonly reported from other countries. The association of campylobacteriosis with consumption of bottled water may be the result of confounding with an unknown variable, such as eating more frequently at restaurants or having limited access to treated tap water. Further investigation of the circumstances in which people choose to drink bottled water in Norway may reveal where and why people choose to consume bottled water, particularly whether it is frequently chosen as an alternative to poor quality water sources. However, a 2003 cohort study from Wales identified drinking bottled water as a risk factor for *Campylobacter* infection in multivariable analysis [32]. This exposure had one of the highest attributable fractions at 12%. Bottled water in Norway is regulated under Norwegian Drinking Water Regulation, which outlines the water quality parameters and protocol for routine control testing that must be followed [33]. However, at least one study found that tap water is of higher quality than bottled water, although both meet the required quality parameters [34].

Animal contact has been identified as a risk factor in many previous studies [15, 35]. Both contact with livestock on a farm with livestock and having a dog in the household were associated with campylobacteriosis. Cats and dogs are known to harbor and excrete *Campylobacter* asymptomatically [36, 37] and having a dog in the household was found to have a significant association with illness in the Norwegian case-control studies conducted in 1989–1990 and 1991–1994. Similarly, living on a farm with livestock implies frequent contact with animals, whether it is occupational or recreational. Livestock, such as sheep, cows, pigs and poultry, can also carry and excrete *Campylobacter*, which can subsequently be transmitted to humans through environmental exposure [38, 39]. Occupational exposure to animals and living on a farm have both been previously reported as risk factors for *Campylobacter* [10, 25, 35]. A previous outbreak of campylobacteriosis among children occurred after a visit to a farm, where the same strain of *C*. *jejuni* was isolated from feces of children and lambs [40]. The persistent result that campylobacteriosis is associated with animal contact supports the importance of good hand hygiene, especially after having contact with animals, using the toilet and before eating.

This study was the first nationwide study of exposures associated with *Campylobacter* infection in Norway. Although the findings support the results of previous regional case-control studies, there are several limitations that must be considered. Only laboratory-confirmed cases were included in the study. It is possible that milder cases did not seek healthcare, which could hide patterns of risk factors. Despite efforts to increase the response proportion through reminders, only 55% of cases and 30% of controls participated in the study, which could mean that results are not necessarily representative of the whole Norwegian population. Although the response proportions were lower than wanted, the relatively large size of the study population and the randomly sampled controls of the population strengthen the study results. As younger people and males were underrepresented compared to the general population, it is possible that non-participating cases and controls differed from participating cases and controls and that relevant exposures were obscured. The possibility of recall bias cannot be excluded. The delay between illness onset and interview may have introduced a recall bias, leading to underestimation of risk factors susceptible to recall problems. Conversely, cases may have had a better recollection of exposures than controls and may have been more likely to report consumption of products typically known to be associated with gastroenteritis. Efforts were made to minimize this bias by sending out questionnaires to both cases and controls on an ongoing basis. However, some segments of the population may have been less likely to have a valid postal address than the general population, which is an inherent limitation of the data collection method. This may have led to an underrepresentation of especially mobile groups, such as students or immigrant populations. In addition, the questionnaire was only available in Norwegian, which may also have led to underrepresentation of the non-Norwegian-speaking population.

## Conclusions

*Campylobacter* infections in Norway appear to be associated with several food and environmental sources. The present study corroborates the results of many studies performed in other countries, as well as previous regional studies conducted in Norway. Consumption of poultry and untreated water remain important sources of infection, despite ongoing control efforts. The results of this study justify the need for strengthening education for consumers and food handlers about the risks associated with consuming raw or undercooked chicken and reinforcing good kitchen hygiene practices. The importance of good hand hygiene should not be underestimated, especially after having contact with animals, after handling raw meat, and before eating. The public should also be reminded to avoid consumption of untreated water and to ensure continued vigilance in order to protect and maintain the quality of water from small-scale water supply systems.

## Supporting Information

S1 Table Derivative univariable logistic regression of selected food and water risk factors for campylobacteriosis. (DOCX)
