# Supplementary material for: Risk Factors for Sporadic Domestically Acquired Campylobacter Infections in Norway 2010–2011: A National Prospective Case-Control Study
Source: PLoS One. 2015 Oct 2;10(10):e0139636. doi: 10.1371/journal.pone.0139636 (PMC4592224; doi:10.1371/journal.pone.0139636)
Supplement: S1 Table — (DOCX) [file pone.0139636.s001.docx]

**S1 Table. Derivative univariable logistic regression of selected food and water risk factors for campylobacteriosis**

|  | **Exposure** | **Cases exposed N (%)** | **Controls exposed N (%)** | **OR (95% CI) Crude model** | **OR (95% CI) Final model^#^** |
| --- | --- | --- | --- | --- | --- |
| **Within people who eat chicken** |  |  |  |  |  |
|  | Bought raw and unfrozen | 286 (54) | 389 (50) | 1.18 (0.95, 1.47) | 1.13 (0.89, 1.43) |
|  | Bought raw and frozen | 150 (28) | 317 (41) | 0.57 (0.45, 0.73)*** | 0.56 (0.44, 0.73)*** |
|  | Products like sausages and meatballs | 81 (15) | 151 (19) | 0.76 (0.57, 1.02)+ | 0.79 (0.58, 1.09) |
|  | Ready-made chicken from serving place | 179 (34) | 196 (25) | 1.53 (1.20, 1.94)*** | 1.45 (1.12, 1.88)** |
| **Within people who eat meat that is not fully cooked** |  |  |  |  |  |
|  | Ground beef | 23 (21) | 16 (13) | 1.70 (0.85, 3.42) | 1.66 (0.73, 3.77) |
|  | Hamburgers | 8 (7) | 6 (5) | 1.48 (0.50, 4.40) | 1.47 (0.34, 6.36) |
|  | Chicken/turkey | 50 (45) | 14 (12) | 6.25 (3.19, 12.24)*** | 8.01 (3.62, 17.72)*** |
|  | Lamb/mutton | 2 (2) | 4 (3) | 0.53 (0.10, 2.97) | 0.69 (0.10, 4.82) |
|  | Beef | 21 (19) | 70 (59) | 0.17 (0.09, 0.30)*** | 0.16 (0.08, 0.32)*** |
|  | Pork | 7 (6) | 15 (13) | 0.47 (0.18, 1.20) | 0.40 (0.13, 1.18)+ |
| **Within people who have eaten food made on a barbecue** |  |  |  |  |  |
|  | Fish | 48 (14) | 51 (14) | 0.97 (0.64, 1.49) | 0.98 (0.62, 1.55) |
|  | Hamburgers | 85 (24) | 92 (25) | 0.94 (0.67, 1.33) | 0.95 (0.65, 1.39) |
|  | Chicken/turkey | 109 (31) | 70 (19) | 1.90 (1.34, 2.68)*** | 1.95 (1.34, 2.84)*** |
|  | Lamb/mutton | 16 (5) | 23 (6) | 0.71 (0.37, 1.36) | 0.82 (0.41, 1.68) |
|  | Sausages | 202 (58) | 216 (60) | 0.93 (0.69, 1.25) | 0.85 (0.61, 1.19) |
|  | Beef | 78 (22) | 73 (20) | 1.14 (0.80, 1.63) | 1.29 (0.87, 1.93) |
|  | Pork | 127 (36) | 134 (37) | 0.97 (0.72, 1.32) | 0.85 (0.60, 1.19) |
| **Within people who have had contact with cats or dogs** |  |  |  |  |  |
|  | Dog had diarrhea or vomited | 22 (7) | 10 (2) | 3.27 (1.52, 7.00)** | 3.07 (1.37, 6.85)** |
|  | Dog had raw bones | 14 (4) | 20 (5) | 0.99 (0.49, 1.99) | 0.95 (0.44, 2.03) |
|  | Dog had dried bones | 97 (31) | 145 (33) | 0.92 (0.67, 1.26) | 0.94 (0.68, 1.31) |
|  | Cat had diarrhea or vomited | 10 (5) | 5 (2) | 2.97 (1.00, 8.84)* | 3.40 (1.07, 10.87)* |
|  | Cat used litterbox | 38 (18) | 80 (27) | 0.61 (0.40, 0.95)* | 0.63 (0.39, 1.01)+ |
|  | Cat had diarrhea or vomited | 27 (13) | 46 (15) | 0.82 (0.49, 1.37) | 0.87 (0.49, 1.52) |
|  | Emptied litterbox | 22 (7) | 10 (2) | 3.27 (1.52, 7.00)** | 3.07 (1.37, 6.85)** |

**^#^***Adjusted for: Is male, Age, Number of people in house, County (dummy variables), Education (categorical). Answers for all variables were not available for all participants. Denominators in percentages vary. Significance indicators: 0 *** 0.001 ** 0.01 * 0.05 + 0.1. Indicators based on P values adjusted for multiple testing using Bonferroni correction.*
